# Near‐Infrared Spectroscopy Combined With Skin Impedance for Detection of Skin Cancer in Primary Care

**DOI:** 10.1111/srt.70344

**Published:** 2026-03-12

**Authors:** Maria Fragkou Dragka, Tommy Löfstedt, Magnus Falk

**Affiliations:** ^1^ Department of Health and Department of Dermatology and Venerology Linkoping University Medicine and Caring Sciences Linköping University Linköping Sweden; ^2^ Department of Computing Science Department of Health Umeå University Umeå Sweden; ^3^ Medicine and Caring Sciences, and Kärna primary Healthcare Center Region Östergötland Linkoping University Linköping Sweden

**Keywords:** basal cell carcinoma, diagnostic accuracy, machine learning, melanoma, near infrared spectroscopy, skin impedance, squamous cell carcinoma

## Abstract

**Background:**

The established method in primary care to distinguish skin cancer from benign lesions is clinical examination, with or without dermoscopy. The experience among primary care physicians in assessing skin tumours varies, as does the accessibility to teledermoscopy. To enhance diagnostic performance, improved methods for skin tumour assessment are warranted. The aim of this study was to investigate the diagnostic performance of a non‐invasive method that combines near‐infrared spectroscopy with skin impedance measurement (NIRIMP) to detect skin cancer in primary care.

**Material and Methods:**

NIRIMP measurements were collected prospectively from patients seeking primary care for skin lesion examination. The measurements were compared to the true lesion diagnosis using several machine learning methods, to determine the best machine learning methods to use and to determine the diagnostic performance of NIRIMP in distinguishing skin cancer from benign lesions.

**Results:**

Eighty participants with 109 lesions were included. Among these, 50 skin cancers or in situ cancers were detected: eight melanomas/in situ melanomas, four in situ squamous cell carcinomas, and 38 basal cell carcinomas. The ability of NIRIMP to distinguish any skin cancer/in situ cancer, as illustrated by the area under the receiver operating characteristics curve (AUC), was 0.776 and for melanomas/in situ melanomas alone the AUC was 0.911. When detecting any skin cancer, the AUC was slightly higher for NIR alone (0.826) compared to NIRIMP (0.776), whereas for IMP alone it was slightly lower (0.693).

**Conclusions:**

Near infrared spectroscopy appears to be a promising bioengineering technique to detect skin cancer in primary care settings, of potential benefit for future skin lesion assessment. However, there was no compelling evidence supporting the benefit of adding skin impedance to improve diagnostic performance.

## 1 Introduction

Skin cancer has increased rapidly in Sweden and worldwide over the past decades. Since 1970, the incidence in Sweden has increased by a factor of 10, a factor twice the average per capita compared to the average in Europe during the same time interval [1, 2, 3]. The main reason behind this increase is believed to be excessive sun exposure habits in combination with a dominance of fair‐skinned inhabitants along with an increasing proportion of elderly in the population [4]. For both melanoma and non‐melanoma skin cancer, the prognosis is good if detected at an early stage of the disease.

Variation in diagnostic and dermoscopic skills to assess skin lesions among physicians contributes to high number of referrals to dermatology clinics, unnecessary excisions of benign lesions, and escalating healthcare costs and undesirable patient discomfort [5]. In a health economic study performed in the year 2000, it was estimated that 154 900 excisions of benign melanocytic lesions during that year in Sweden led to 300 million SEK in healthcare costs (with today's value corresponding to approximately 475 million SEK/42,5 million EUR) [6, 7]. The use of dermoscopy has been shown to improve diagnostic performance among trained users, but requires substantial training and experience to perform [8]. To be able to meet the healthcare needs of the population, novel methods to assist in distinguishing malignant from benign skin lesions are warranted to facilitate diagnostics and potentially improve the quality of care. Methods that have shown promising results include optical spectroscopy and electrical impedance measurement [9, 10, 11, 12, 13]. Of these, variants of optical spectroscopy has in performed studies, based on a systematic review and meta‐analysis from 2021, scored highest with regards to diagnostic performance levels (average sensitivity and specificity of 93% and 82.2%, respectively) and somewhat lower for electrical skin impedance measurement (83%–97% sensitivity and 30%–68% specificity in detecting melanoma [14]. In recent studies, not covered by the review, elastic‐scattering spectroscopy and Raman spectroscopy have in recent studies showed promising levels of diagnostic performance in differentiating skin cancers from benign lesions [15, 16, 17].

A few studies have investigated the joint diagnostic performance of combining near infrared spectroscopy with electrical skin impedance (hereafter denoted NIRIMP), indicating that the two methods together could successfully be used to differentiate between melanomas and benign nevi, and at various anatomic body sites, with a higher precision than either method alone [11, 13]. In a first study, NIRIMP presented with a sensitivity of 83 % and specificity of 95% in detecting melanoma. In a following prospective pilot study performed in primary, NIRIMP was used to distinguish skin cancer (both melanoma and non‐melanoma) from benign lesions in a sample of 38 skin tumours, with 100% sensitivity and 100% specificity [11]. Although promising, with regards to this result, the study suffered from a limited sample size, in part associated with a quite substantial number of drop‐outs due to measurement errors. As a method, NIRIMP has the advantage of being non‐invasive, potentially cheap, and readily available compared to many other diagnostic techniques for skin cancer assessment [11, 13]. However, further investigations are needed, with larger samples of patients, and with both melanocytic and non‐melanocytic lesions. The aim of this study was therefore to further investigate the diagnostic ability of NIRIMP in distinguishing skin cancer from benign lesions assessed in primary care.

## 2 Materials and Methods

### 2.1 Study Population

This study was part of a real‐life prospective multicentre study in Sweden during 2017–2021, exploring the diagnostic performance of NIRIMP in primary care and at dermatology clinics, after approval by the Regional Ethical Review Board in Linköping (Dnr. 2017/176‐31). The study protocol is registered at the ISRCTN database (no. 36618), and performed and reported in concordance with the STARD guidelines.

The present paper addresses the primary care part of the project, which took place at Kärna primary care centre in Linköping, Sweden, located in Southeast Sweden and serving a population of approx. 11.500 inhabitants. Patients above 18 years of age seeking the primary care centre for examination of skin lesions of concern were considered for inclusion. Recruitment was performed in conjunction with a doctor's consultation for assessment of the patient's skin lesions, and if the patient had at least one skin lesion that the examining doctor could not confidently dismiss as benign, the patient was eligible for inclusion. The following inclusion criteria were applied: (1) At least one, skin lesion suspected to be malignant (independently of type), (2) Either pigmented or non‐pigmented lesions, and (3) Skin types I–IV according to Fitzpatrick's classification. Inability to provide informed consent, nodular lesions, lesions located on the palms, on the head and areas with dense hair growth were exclusion criteria. The exclusion of lesions on these locations was due to difficulties to provide reliable and reproducible measurements, as based on the experience from previous studies [11, 13]. All participants provided written informed consent before being included in the study.

### 2.2 Diagnosis Retrieval

Since all included lesions were associated with some degree of suspicion of skin cancer, the patients underwent standard clinical investigation of the lesion/s, either by excision, by skin biopsy or by referral to dermatologist, in strict concordance with to the national clinical guidelines for the management of skin tumour diagnostics (i.e., being the gold standard in the studied setting). If referred to dermatologist and the dermatologist judged a lesion to be undoubtedly benign, without need for histopathological confirmation, the final lesion diagnosis was based solely on the dermatologist assessment. In addition, in clinically doubtless cases of superficial BCC lesions receiving other treatments than surgical excision, such as cryotherapy or field therapy not generating any biological material for histopathological analysis, histopathology is normally not performed as part of standard management. In all other cases, the diagnoses were set by histopathologic analysis, regardless of whether excised/biopsied in primary care or by the dermatologist after referral. Importantly, this procedure complies to the standard clinical routine for managing skin lesions in the study setting, that is, Swedish primary care. The dermatologists and pathologists determining the diagnoses were neither involved, nor informed about the patients being part of the study, in order to secure an unbiased diagnostic process not deviating from standard procedures. Retrieval of tumour diagnoses were performed from the participants’ electronic patient records, by permission from the patients.

### 2.3 Study Design

Study participants of both sexes were included consecutively when attending the primary care centre (see Table 1 for distribution). During the physical examination, the doctor determined whether the patients’ lesions were either by certainty benign (and thus not to be included), or at least to some degree suspected to be malignant (melanoma or non‐melanoma skin cancer). If fulfilling inclusion criteria, the patients were then asked to participate.

**TABLE 1:** Distribution of lesion diagnoses in the study population, including diagnosis method.

Lesion diagnosis	Number (n)			Diagnosis method (n)	
	Men	Women	Total	Histopathology	Dermatologist assessment
Melanoma	3	2	5	5	0
Melanoma in situ	3	0	3	3	0
Basal cell carcinoma	21	17	38	29	9
Squamous cell carcinoma in situ	3	1	4	4	0
Actinic keratosis	13	3	16	4	12
Dysplastic nevus	4	0	4	4	0
Melanocytic nevus (non‐dysplastic)	5	9	14	8	6
Seborrhoeic keratosis	6	6	12	9	3
Other Benign lesions	9	4	13	7	6

After study inclusion, NIRIMP measurements were undertaken on the suspected skin lesions. For practical reasons (time needed to be allocated to perform the measurements) a maximum of three lesions per patient could be included. For each lesion, three measurements were performed on the lesion and three on unaffected skin at the corresponding location at the contralateral side of the body. The total measurement time for each lesion was 5–10 min.

**FIGURE 1:**
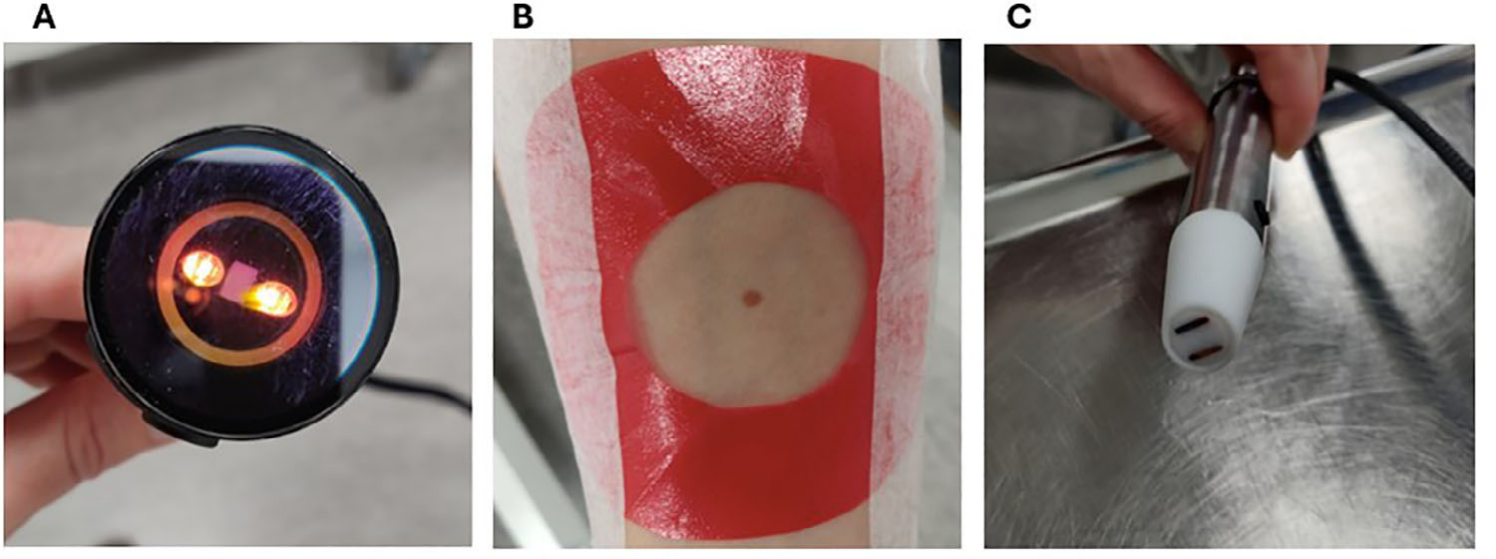
Photos illustrating (A) the NIR spectroscope head, (B) the template used for centration of the NIR spectroscope head on the measurement skin area, and (C) the IMP probe.

The NIR‐spectra were measured using a NIR Spectrometer (MicroNIR 1700ES, Viavi, Chandler, AZ, USA; see Figure 1A) connected to a PC laptop with associated software (Viavi MicroNIR Pro, Chandler, AZ, USA). A template was used for both the skin lesion and reference point measurements (see Figure 1B) to fit the active area of the spectrometer correctly over the suspected lesion and at the exact same area for each measurement. The result of the measurement yielded pseudo‐absorbencies at 125 wavelengths between 960–1650 nm. The PC laptop was connected to a probe to measure the electrical impedance (see Figure 1C) and the corresponding software LABVIEW (National instruments AB Stockholm, Sweden). To decrease the resistance in the skin it was soaked with a saline solution (NaCl 0.9%) for 90 s prior to the measurement. The air pressure was reduced to 750 mbar to improve the contact between the skin and the probe. This created small marks on the skin that were used as reference points in the following measurements. LABVIEW collected the measurements of phase shift, Ф, and the magnitude |Z| at 50 frequencies logarithmically spaced between 0.1 and 10 MHz. The values were then transformed into the complex impedance with a real and imaginary part as,

$$I=\left|Z\right|\cdot \;e^{j\Phi }=Re\left(Z\right)+j\cdot Im\left(Z\right),$$

in which,

$$Re\left(Z\right)=\left|Z\right|\cdot \cos\left(\Phi \right)\;\mathrm{and}\;Im\left(Z\right)=\left|Z\right|\cdot \sin\left(\Phi \right).$$

For more details regarding the instruments used the reader is referred to Bodén et al. [13].

Following the measurement procedure, the participants underwent standard diagnostic investigation, as described above. The results from the NIRIMP measurements were compared to the diagnosis set by this standard diagnostic procedure.

### 2.4 Data Analysis

Three sets of data were assembled for analyses: The NIR data by itself (NIR), the impedance data by itself (IMP), and the NIR and IMP data merged (NIRIMP). For each measurement, we considered the lesion alone (labelled LESION) and the difference between the lesion and its control site on the contralateral side (labelled LESION vs. CONTROL). The data were analysed with respect to three labels: Whether the lesion was cancerous/in situ cancerous (labelled ANY CANCER), whether it was a melanoma/in situ melanoma (labelled MELANOMA), and whether it was a pigmented lesion or not (labelled PIGMENTED LESION). The lesions were analysed with respect to the ANY CANCER and MELANOMA labels, and the MELANOMA label was analysed for patients restricted to having a positive PIGMENTED LESION label. In both cases analyses were made for differences between the lesion and its control, but also for the lesion alone, with respect to the ANY CANCER (i.e., any skin cancer/in situ cancer type) and MELANOMA (i.e., strictly melanoma/in situ melanoma) labels. We kept 10% of the data until the end of the study as hold‐out test set, that was used to give a final indication of how the methods generalised to new data.

We performed a comprehensive comparison of four different machine learning methods, including non‐linear methods such as support vector machines and neural networks, to robustly evaluate the data. The chosen methods all represent standard methodology with proven and well‑documented good predictive ability. The three data sets were analysed using the following machine learning methods: Logistic regression with elastic net regularisation (LR), a linear support vector machine (with a squared hinge loss and a ridge penalty; Linear SVM), a non‐linear support vector machine (with a radial basis kernel, RBF; Non‐linear SVM), random forest (RF), and a fully connected neural network model (NN). The neural network model was implemented using Tensorflow and Keras, while the implementations in scikit‐learn were used for the other models.

Each machine learning method had several hyper‐parameters that had to be found. We used Bayesian optimisation for the NN model using Gaussian process regression for 30 iterations using the expected improvement criterion [18]. Grid search was used for all other methods. Ten‐fold cross‐validation (CV) was used, with stratification on the patients (to have repeated measurements from the same patients in either train or test sets, but not in both). The criterion optimized over was the area under the receiver operating characteristic (ROC) curve (AUC), and ties were broken using the highest Matthew's correlation coefficient (MCC). Accuracy (ACC) and positive predictive values (PPV) were also computed.

We also evaluated whether there were issues with class imbalance by training all models using reciprocal frequency weighting and training them without considering such issues. We further investigated whether applying standard scaling (subtracting the mean and normalising to unit variance) improved the results or not.

The hyper‐parameters for each method were:

- Logistic regression: Regularisation strength (11 values on a log‐scale between 0.001 and 10) and the radio between the L1 and L2 penalties (11 values linearly between 0 and 1).
- Linear SVM: Regularisation strength (31 values on a log scale between 0.01 and 10).
- Non‐linear SVM: Regularisation strength (11 values on a log‐scale between 0.01 and 1000) and the kernel length scale (11 values on a log‐scale between 10^−7^ and 1).
- Random forest: The number of trees (between 40 and 200 in steps of 40), the variable split criterion (gini index or information gain), the trees’ maximum depth (no limit, or values between 2 and 18 in steps of 4), and the minimum number of samples to split a node (4 or 8).
- Neural network: Number of layers (uniformly between 1 and 3), the number of units in each layer (uniformly at random between 8 and 128), whether to use batch normalization or not, the dropout rate (uniformly between 0.01 and 0.99), the learning rate (between 10^−5^ and 1), the mini‐batch size (between 8 and 64), the number of training epochs (between 50 and 1000), and the L2 regularization strength (uniformly on a log‐scale between 10^−7^ and 1).

Note that both the imbalanced weighting and the standard scaling were also hyper‐parameters evaluated (that were used or not). Note also that some ranges are very wide, for instance the kernel length scale, the learning rate, and the L2 regularization strength. The reason for these hyper‐parameter ranges to be so wide was because they are unknown and that the authors know from experience that they can be both very small and relatively large in applications. Further, we attempted to make the ranges go through even factors of 10 for simplicity and that the L2 regularization should be allowed to be very small (almost non‐existent).

After finding the best hyper‐parameter combination for each model, we performed another round of 10‐fold CV for all models using the found best set of hyper‐parameters. We also applied the models to the held‐out test data set and used the Bootstrap to compute the standard errors for the test results. The 10‐fold CV thus better accounts for variability in the different folds because of small numbers of validation samples, but is biased because it was also used for model selection; the test data, on the other hand, provides an unbiased estimate of the performance, but one that has a high variability.

The significances of the results were determined using standard significance thresholds for the three metrics, set at 0.5 for AUC, at zero correlation for MCC, and at zero for PPV. For accuracy (the proportion of correctly predicted lesion outcomes) we computed the base rate (the maximum of the fraction of positive and negative labels) as the mean base rate from a stratified 10‐fold CV (stratified on the patients, so that all data from the same patient would end up in the same splits to avoid data leakage). Then we set the significance threshold to the mean base rate frequency for the majority class. We used three significance levels in the presentation of the results, with one, two, or three standard errors above the base rate thresholds, and indicated the level by that number of star asterisks.

We performed a Friedmann test of equivalence to determine if the results were different when using the different data sets (NIR, IMP, or NIRIMP). We used the AUC results from all evaluated methods, and performed the test separately for skin cancer/in situ cancer, melanoma/in situ melanoma, and melanoma/in situ melanoma exclusively among pigmented lesions (i.e., the tests were distinguished like the results in Tables 2 and 3 below). In case of a *p* value from the Friedman test below 0.05, we performed a Nemenyi post‐hoc test to compare the pairwise differences between the methods [19].

**TABLE 2:** Diagnostic performance of NIR, IMP, and NIRIMP to detect any skin cancer/in situ cancer (A) and melanoma/in situ melanoma (B), respectively.

*A. Any skin cancer*							
		Any cancer/in situ cancer based on lesion alone			Any cancer/in situ cancer based on lesion versus control		
Measurement technique	Metric	Method	Result (CV)	Result (hold‐out test)	Method	Result (CV)	Result (hold‐out test)
NIR	AUC	NN^bn^	0.755 (0.034) ***	0.467 (0.099)	RF^bn^	0.826 (0.031) ***	0.643 (0.165)
	ACC	RF^n^	0.672 (0.050) ***	0.583 (0.082) *	SVM (linear)^n^	0.737 (0.051) ***	0.667 (0.136) *
	MCC	NN^bn^	0.369 (0.066) ***	−0.068 (0.171)	SVM (RBF)^bn^	0.485 (0.042) ***	0.378 (0.271) *
	PPV	RF^n^	0.674 (0.060) ***	0.500 (0.107) ***	SVM (linear)^n^	0.775 (0.078) ***	0.556 (0.166) ***
IMP	AUC	SVM (RBF)^bn^	0.674 (0.048) ***	0.628 (0.098) *	NN	0.693 (0.038) ***	0.500 (0.183)
	ACC	NN^n^	0.650 (0.031) ***	0.667 (0.082) **	NN^b^	0.675 (0.054) ***	0.545 (0.150)
	MCC	NN^n^	0.308 (0.066) ***	0.332 (0.160) **	NN^b^	0.346 (0.117) **	0.069 (0.332)
	PPV	NN^n^	0.649 (0.049) ***	0.625 (0.121) ***	LR^bn^	0.665 (0.065) ***	0.333 (0.272) *
NIRIMP	AUC	NN^bn^	0.782 (0.045) ***	0.567 (0.180)	SVM (RBF)^bn^	0.776 (0.037) ***	0.900 (0.100) ***
	ACC	NN^n^	0.703 (0.056) ***	0.545 (0.150)	NN^n^	0.731 (0.039) ***	0.818 (0.116) ***
	MCC	NN^bn^	0.437 (0.083) ***	0.149 (0.326)	NN^n^	0.484 (0.074) ***	0.633 (0.200) ***
	PPV	NN^n^	0.705 (0.074) ***	0.500 (0.177) **	NN^n^	0.760 (0.056) ***	0.800 (0.179) ***

*B. Melanoma/in Situ melanoma*							
		Melanoma/in situ melanoma based on lesion alone			Melanoma/in situ melanoma based on lesion versus control		
Measurement technique	Metric	Method	Result (CV)	Result (hold‐out test)	Method	Result (CV)	Result (hold‐out test)
NIR	AUC	NN^bn^	0.859 (0.076) ***	0.652 (0.152)	SVM (RBF)^n^	0.956 (0.023) ***	0.500 (0.314)
	ACC	SVM (linear)^n^	0.930 (0.015) ***	0.917 (0.046) ***	SVM (linear)^n^	0.930 (0.015) ***	0.917 (0.080) ***
	MCC	NN	0.348 (0.142) **	0.000 (0.171)	LR^b^	0.388 (0.133) **	−0.135 (0.310)
	PPV	NN^bn^	0.339 (0.138) **	0.115 (0.063) *	LR^b^	0.350 (0.118) **	0.000 (0.000)
IMP	AUC	SVM (RBF)^n^	0.807 (0.064) ***	0.500 (0.176)	SVM (RBF)^n^	0.897 (0.067) ***	0.500 (0.314)
	ACC	LR^n^	0.928 (0.015) ***	0.917 (0.046) ***	LR^n^	0.928 (0.015) ***	0.917 (0.080) ***
	MCC	LR^n^	0.300 (0.145) **	0.000 (0.171)	LR^n^	0.300 (0.145) **	0.000 (0.316)
	PPV	LR^bn^	0.339 (0.137) **	0.000 (0.000)	LR^n^	0.300 (0.145) **	0.000 (0.000)
NIRIMP	AUC	NN	0.837 (0.068) ***	0.500 (0.314)	SVM (linear)^n^	0.911 (0.041) ***	0.500 (0.314)
	ACC	LR^n^	0.928 (0.015) ***	0.917 (0.080) ***	RF^n^	0.928 (0.015) ***	0.917 (0.080) ***
	MCC	LR^n^	0.300 (0.145) **	0.000 (0.316)	SVM (linear)^n^	0.423 (0.165) **	0.000 (0.316)
	PPV	NN^b^	0.332 (0.139) **	0.083 (0.080) *	SVM (linear)^n^	0.450 (0.149) ***	0.000 (0.000)

*Note*: A superscript *n* indicates that the best model used normalisation (standard scaling), and a superscript *b* indicates that the best model used reciprocal frequency weighting. The number of star asterisks indicates one, two, or three standard errors above the base rate thresholds, respectively.

**TABLE 3:** Diagnostic performance of NIRIMP to detect melanoma/in situ melanoma exclusively among pigmented lesions. A superscript *n* indicates that the best model used normalisation (standard scaling), and a superscript *b* indicates that the best model used reciprocal frequency weighting. The number of star asterisks indicates one, two, or three standard errors above the base rate thresholds, respectively.

Measurement technique	Metric	Melanoma/in situ melanoma based on lesion alone			Melanoma/in situ melanoma based on lesion versus control		
		Method	Result (CV)	Result (hold‐out test)	Method	Result (CV)	Result (hold‐out test)
NIR	AUC	SVM (RBF)^n^	0.825 (0.064) ***	0.500 (0.188)	SVM (RBF)^n^	0.933 (0.063) ***	0.500 (0.342)
	ACC	LR^n^	0.835 (0.035) ***	0.833 (0.088) ***	RF^bn^	0.885 (0.037) ***	0.833 (0.152) ***
	MCC	NN	0.331 (0.140) **	−0.200 (0.240)	RF^bn^	0.619 (0.137) ***	0.000 (0.500)
	PPV	NN	0.349 (0.138) **	0.000 (0.000)	RF^bn^	0.600 (0.138) ***	0.000 (0.000)
IMP	AUC	SVM (RBF)^n^	0.817 (0.082) ***	0.500 (0.188)	LR^n^	0.883 (0.075) ***	0.500 (0.342)
	ACC	LR^n^	0.835 (0.035) ***	0.833 (0.088) ***	LR^bn^	0.905 (0.037) ***	0.667 (0.192) **
	MCC	NN^b^	0.330 (0.123) **	0.000 (0.250)	LR^bn^	0.600 (0.155) ***	−0.200 (0.480)
	PPV	NN^b^	0.438 (0.127) ***	0.000 (0.000)	LR^bn^	0.600 (0.155) ***	0.000 (0.000)
NIRIMP	AUC	NN	0.825 (0.112) **	0.500 (0.342)	NN	0.908 (0.065) ***	0.900 (0.157) **
	ACC	NN	0.860 (0.037) ***	0.833 (0.152) ***	LR^bn^	0.905 (0.037) ***	0.667 (0.192) **
	MCC	NN	0.400 (0.155) **	0.000 (0.500)	LR^bn^	0.600 (0.155) ***	−0.200 (0.480)
	PPV	NN^b^	0.425 (0.150) **	0.000 (0.000)	LR^bn^	0.600 (0.155) ***	0.000 (0.000)

## 3 Results

**FIGURE 2:**
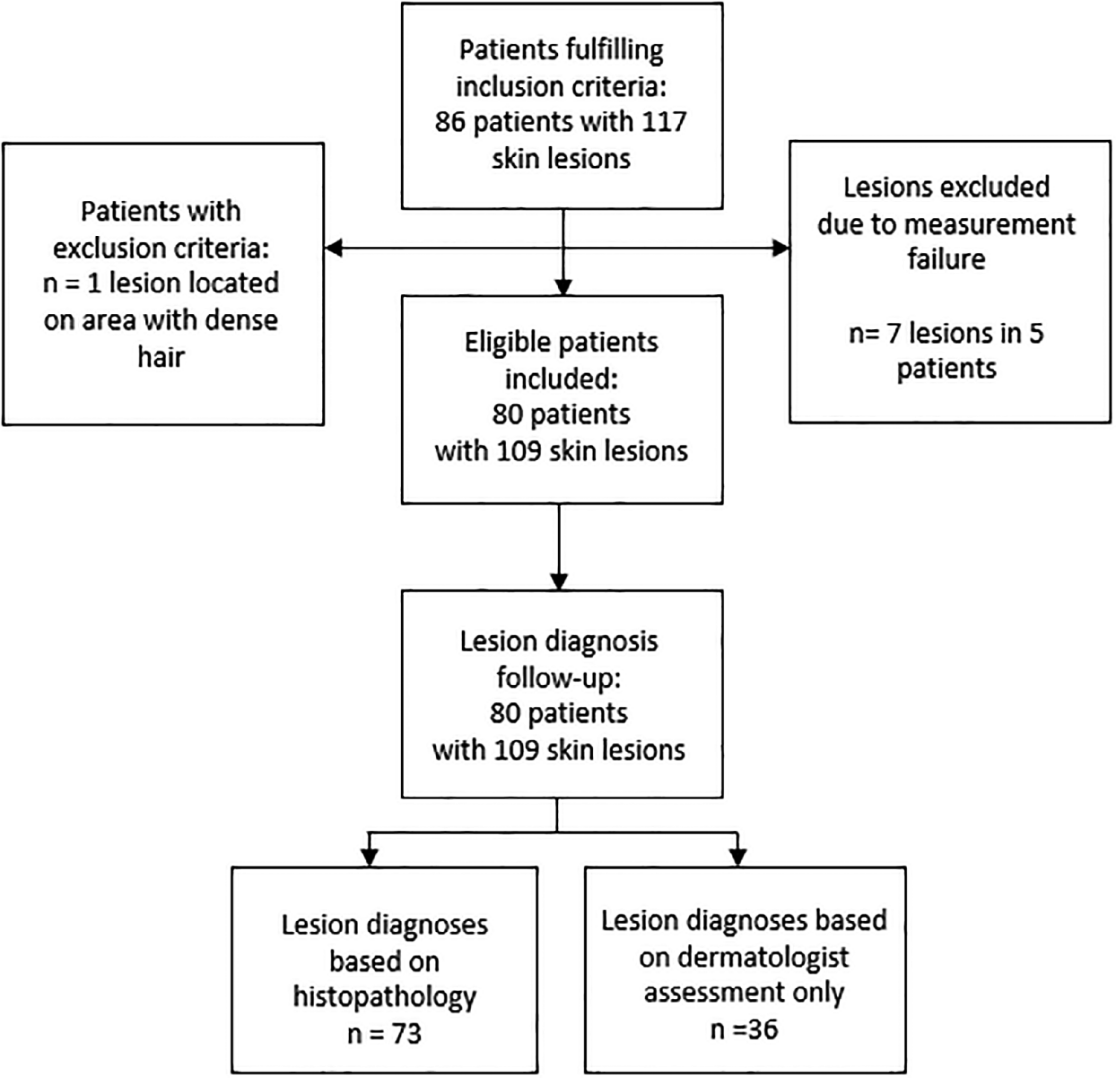
Participant flowchart.

A total of 86 participants with 117 lesions were recruited. Of these, eight lesions in six participants were excluded due to measurement errors (NIR or IMP measurement missing or incomplete, in one case due to lesion location in dense hair, that is, exclusion criterion), see flowchart (Figure 2). Consequently, 80 participants were included in the study, with a total of 109 lesions. The participants’ ages varied from 20 to 88 years. Most of the participants (53 %) were between 61 and 80 years old, 21% between 41–60 years, 15% between 81–100 years and 11% between 20–40 years. Table 1 presents the distribution of lesion diagnoses determined by the regular diagnostic investigation. For 73 (67%) of the 109 lesions, the diagnosis was based on histopathology, whereas for the remaining 36 lesions (33%) it was based om dermatologist assessment only.

### 3.1 Diagnostic Accuracy for NIRIMP

**FIGURE 3:**
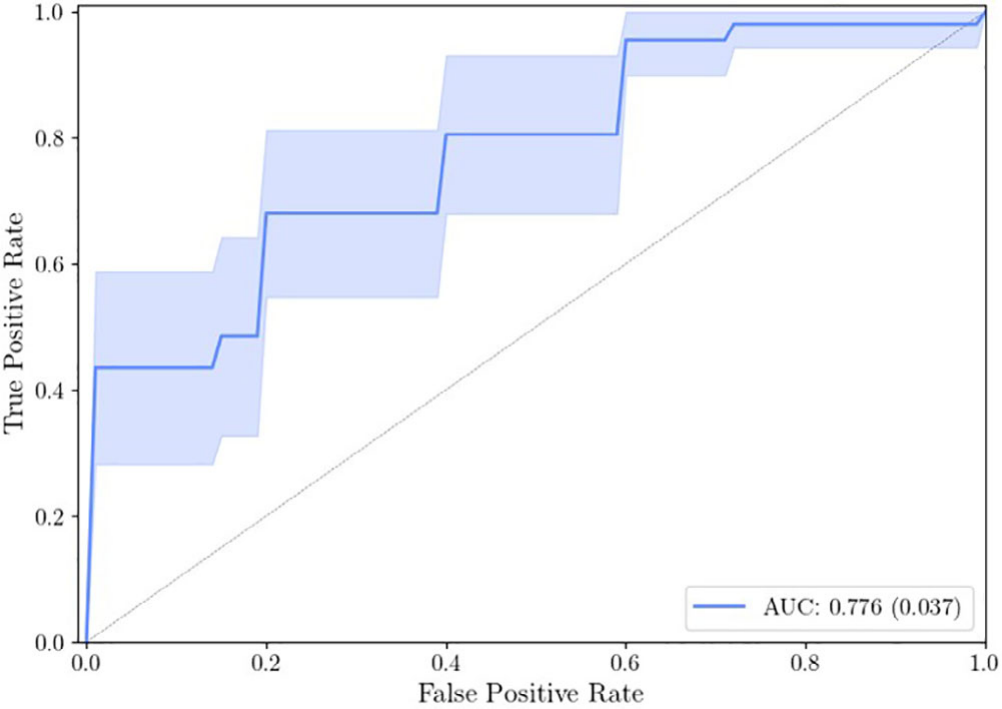
ROC curve illustrating the diagnostic performance of NIRIMP to detect any skin cancer/in situ cancer (melanoma and non‐melanoma), including the area under the curve (AUC).

**FIGURE 4:**
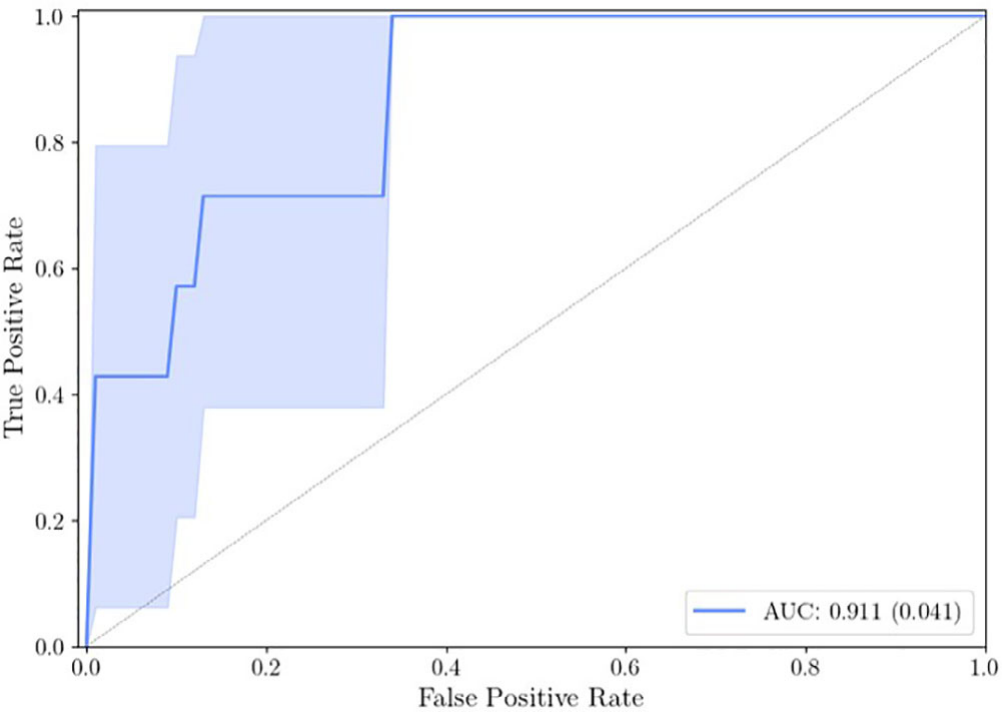
ROC curve illustrating the diagnostic performance of NIRIMP to detect melanoma skin cancer/in situ only, including the area under the curve (AUC).

In Figure 3, the diagnostic performance of NIRIMP to detect any skin cancer (melanoma and non‐melanoma) is presented in a ROC (receiver operating characteristics) curve, the AUC being 0.776. As illustrated in the curve, at a true positive rate of 0.8 (or 80% sensitivity), the corresponding false positive rate was 0.4, equalling 60% specificity (1 – false positive rate). Similarly, at 63% sensitivity, the specificity increases to 80% (i.e., 0.2 false positive rate). When restricting the analysis exclusively to address melanoma and melanoma in situ, the corresponding AUC increased to 0.911, at best corresponding to 63% specificity at a 100% sensitivity level (Figure 4).

The diagnostic performance of NIRIMP and its respective components (NIR and IMP) are further explored in more detail in Table 2, and displayed as AUC, ACC, MCC, and PPV, respectively. In addition, besides the outcome for lesions when compared to its control, the corresponding outcome for the lesion alone (i.e., without comparison with its control) is also presented. As can be seen, AUC for NIR alone to detect any skin cancer/in situ cancer was slightly higher (0.826) than for NIRIMP combined (0.776), whereas for IMP alone it was slightly lower (0.693). Targeting melanoma, the highest AUC level was seen for NIRIMP combined (0.911). However, the very highest AUC value to detect melanoma/in situ melanoma was shown for NIR alone (0.956). The outcome for IMP alone was generally lower in all respects, compared with both NIRIMP and with NIR alone.

Table 3 presents the diagnostic performances to detect melanoma/in situ melanoma when restricted to pigmented lesions. The outcome for all three entities (NIRIMP, NIR, and IMP) were more consistent, and in all cases slightly higher when comparing the lesion with its control.

The Friedman test for skin cancer/in situ cancer returned a *p* value of 0.0006, for melanoma/in situ melanoma it was 0.002, and for melanoma/in situ melanoma exclusively among pigmented lesions it was 0.18. Hence, we cannot say that there are significant differences for melanoma/in situ melanoma exclusively among pigmented lesions, but we can do that for skin cancer/in situ cancer and for melanoma/in situ melanoma. The Nemenyi test for skin cancer/in situ cancer returned the *p* values shown in Table 4a. As can be seen, there were significant differences between NIR and IMP and between NIR and NIRIMP, but we could not detect a difference between IMP and NIRIMP. In Table 4b, the corresponding *p* values for melanoma/in situ melanoma returned the *p* values are presented. As shown, there were significant differences between NIR and NIRIMP, we did not have a clear difference between NIR and IMP (no difference on the 5% level, but there is a difference on the 10% level), and there was no difference between IMP and NIRIMP.

**TABLE 4:** *p* values obtained by Nemenyi test for (A) skin cancer/in situ cancer and (B) melanoma/in situ melanoma, illustrated for NIR, IMP and NIRIMP, respectively.

A				B			
	NIR	IMP	NIRIMP		NIR	IMP	NIRIMP
NIR	—	0.01	0.01	NIR	—	0.07	0.02
IMP	0.01	—	0.76	IMP	0.07	—	0.86
NIRIMP	0.01	0.76	—	NIRIMP	0.02	0.86	—

Through the evaluation of machine learning methods, it was found that the results are model‐dependent. When maximizing AUC, the most common best performing methods were neural networks and non‐linear support vector machines. Random forest and logistic regression also performed the best occasionally.

## 4 Discussion

In this prospective clinical study, performed in an authentic primary care setting, addressing skin lesions of concern, we found an overall moderate levels of diagnostic performance of NIRIMP in distinguishing skin cancers and in situ cancers (including both melanocytic and non‐melanocytic lesions) from other skin tumours. The best performance was seen when measuring both on the suspected lesion and a control skin site, as previously described for the method, but even when using solely the suspected lesion, accuracy levels were still almost as high. However, since higher levels of diagnostic accuracy were seen for NIR alone, the value of adding skin impedance to the assessment does not in the primary analyses emerge as beneficial as has previously been found by Bodén et al., illustrating an additive increase in diagnostic performance by combining the two methods [13, 20]. On the other hand, NIRIMP combined and measured on both the lesions and its controls to detect any skin cancer/in situ cancer (Table 2A) was the measurement technique performing best in the hold‐out test. The moderate sample size, and especially the limited number of melanomas, is likely to have contributed to the overall moderate outcome, especially when considering the markedly better performance to specifically detect melanomas/in situ melanomas than any cancer/in situ cancer, in this respect, reaching AUC above 0.9. The results do vary somewhat, and there is sometimes a discrepancy between the cross‐validation and hold‐out test results. Since we regularise the models heavily, we conclude that the reason for this is likely that the test data are not fully representative of the population. This further suggests a larger study would be warranted, to give both larger training data but importantly a larger test set to evaluate the generalisation performance. Since the present study had a relatively large cohort, compared to the cohort sizes common in the literature, this may carry a broader message to the field about the importance of larger cohort sizes. Larger future studies would thus be imperative to perform to determine the diagnostic value of NIRIMP with more confidence.

The results of this study are partly consistent with previous findings on NIR and/or IMP. Although studies on NIR alone are scarce, sensitivity and specificity levels of 78.6% and 84.6%, respectively, have been reported [21]. For IMP alone, the scientific basis is somewhat more extensive, quite consistently indicating high levels of sensitivity, but less favourable levels of specificity regarding both melanoma [9, 10, 14, 22, 23], as well as keratinocyte cancer detection [22]. Regarding light spectroscopy, different kinds of multispectral imaging devices to detect skin cancer have also been evaluated, however with no obvious diagnostic advantage in comparison to NIR [24, 25]. However, it might be interesting to combine different types of spectroscopy methods, such as multispectral, Raman, and multispectral, scattering spectroscopy [14, 15, 16, 17], with NIR to investigate potential additive performance effects.

The strength of this study is that it was performed in a real‐life primary care clinical setting, with consecutive patients seeking their primary care physician for skin lesions of concern, prospectively included as study participants, following standard diagnostic procedure. This contributes to the authenticity of the study, as well as to the applicability of its results. Consequently, a natural limitation of the chosen study design is the absence of histologically verified diagnoses for some of the lesions classified as benign. It is well known that dermatologists do not perform 100% diagnostic accuracy when compared to histopathology. However, as pointed out, this limitation in fact reflects the standard diagnostic procedure in Swedish primary care, that is, that skin lesions determined as undoubtedly benign by the dermatologist do not proceed to histopathological assessment. Thus, in reality, these would never be subject to histopathological analysis, why adopting a diagnostic protocol that mandates histopathological assessment of every lesion would constitute a departure from standard clinical practice in primary care and would therefore not represent real‑world diagnostic pathways for skin tumours. Moreover, histopathological investigation for the mere sake of the study would not have been ethically justifiable and would by high likeliness also have led to unfortunate drop‐outs and consequent selection bias. However, just as importantly, the results of the present study are not to be applied on any other clinical settings, such as secondary care.

Regarding pigmented lesions, the restricted sub‐analysis made for melanoma/in situ melanoma detection are of specific relevance due to the common differential diagnostic challenges these often represent in the clinical situation, although it is important to denote that pigmentation occurs also in non‐melanoma skin cancer, and that absence of pigmentation does not exclude melanoma. From a differential diagnostic perspective, it would likewise have been of relevance to specifically investigate the ability of NIRIMP to restrictedly distinguish between squamous skin carcinoma and actinic keratoses (or even other hyperkeratotic benign lesions in general). However, this remains to be explored. As pointed out, an important study limitation in this respect is the relatively small population sample size, but also the lack of squamous cell carcinomas (besides in situ) in the sample and that it was a single‐centre study. As it stands, we acknowledge that these results may be underpowered. To increase generalizability, as well as allowing for further sub‐population analyses of relevance, a larger, multi‐centre study would be necessary. Another limitation regarding the clinical applicability of NIRIMP is the practical difficulty of applying it on lesions located, for example, on the face and hands, particularly since these constitute a common location for skin cancers. Technical improvements of the NIR and IMP probes allowing for better accessibility on irregular shaped skin areas would thus be beneficial.

## 5 Conclusion

Near infrared spectroscopy appears to be a potentially useful method in distinguishing skin cancers from benign lesions, on the primary care level. The value of combining it with skin impedance to increase the diagnostic performance is uncertain, but performed well in the hold‐out test, indicating a potential benefit. Although highly promising, the technique needs further development and evaluation in even larger study populations, ideally with a predefined threshold based on its best performance in this study, to determine its diagnostic reliability and practicability.

## Funding

The study was financed by fundings from FORSS (The Research Council of Southeast Sweden), and by Region Östergötland.

## Consent

Informed consent was obtained from all subjects involved in the study.

## Ethics Statement

The study was conducted according to the guidelines of the Declaration of Helsinki and approved by the Regional Ethical Review Board in Linköping, Dnr. 2017/176‐31.

## Conflicts of Interest

The authors declare no conflict of interest.

## Data Availability

The data that support the findings of this study are available from the corresponding author upon reasonable request.
